# Melatonin-Producing *Bacillus aerius* EH2-5 Enhances *Glycine max* Plants Salinity Tolerance Through Physiological, Biochemical, and Molecular Modulation

**DOI:** 10.3390/ijms26167834

**Published:** 2025-08-13

**Authors:** Eun-Hae Kwon, Suhaib Ahmad, In-Jung Lee

**Affiliations:** 1 Department of Applied Biosciences, Kyungpook National University, Daegu 41566, Republic of Korea; ekwon4@cougarnet.uh.edu; 2 Department of Engineering Technology, Cullen College of Engineering, University of Houston, Houston, TX 77479, USA; sahmad35@cougarnet.uh.edu; 3 Department of Biology and Biochemistry, College of Natural Science and Mathematics, University of Houston, Houston, TX 77479, USA

**Keywords:** melatonin, *Bacillus aerius*, salinity stress, soybean, melatonin producing microbes, PGPB, abiotic stresses, WGS

## Abstract

Climate change has intensified extreme weather events and accelerated soil salinization, posing serious threats to crop yield and quality. Salinity stress, now affecting about 20% of irrigated lands, is expected to worsen due to rising temperatures and sea levels. At the same time, the global population is projected to exceed 9 billion by 2050, demanding a 70% increase in food production (UN, 2019; FAO). Agriculture, responsible for 34% of global greenhouse gas emissions, urgently needs sustainable solutions. Microbial inoculants, known as “plant probiotics,” offer a promising eco-friendly alternative by enhancing crop resilience and reducing environmental impact. In this study, we evaluated the plant growth-promoting (PGP) traits and melatonin-producing capacity of *Bacillus aerius* EH2-5. To assess its efficacy under salt stress, soybean seedlings at the VC stage were inoculated with EH2-5 and subsequently subjected to salinity stress using 150 mM and 100 mM NaCl treatments. Plant growth parameters, the expression levels of salinity-related genes, and the activities of antioxidant enzymes were measured to determine the microbe’s role in promoting plant growth and mitigating salt-induced oxidative stress. Here, our study shows that the melatonin-synthesizing *Bacillus aerius* EH2-5 (7.48 ng/mL at 24 h after inoculation in Trp spiked LB media) significantly improved host plant (*Glycine max* L.) growth, biomass, and photosynthesis and reduced oxidative stress during salinity stress conditions than the non-inculcated control. Whole genome sequencing of *Bacillus aerius* EH2-5 identified key plant growth-promoting and salinity stress-related genes, including znuA, znuB, znuC, and zur (zinc uptake); ptsN, aspA, and nrgB (nitrogen metabolism); and phoH and pstS (phosphate transport). Genes involved in tryptophan biosynthesis and transport, such as trpA, trpB, trpP, and tspO, along with siderophore-related genes yusV, yfhA, and yfiY, were also detected. The presence of multiple stress-responsive genes, including dnaK, dps, treA, cspB, srkA, and copZ, suggests EH2-5′s genomic potential to enhance plant tolerance to salinity and other abiotic stresses. Inoculation with *Bacillus aerius* EH2-5 significantly enhanced soybean growth and reduced salt-induced damage, as evidenced by increased shoot biomass (29%, 41%), leaf numbers (12% and 13%), and chlorophyll content (40%, 21%) under 100 mM and 150 mM NaCl compared to non-inoculated plants. These results indicate EH2-5′s strong potential as a plant growth-promoting and salinity stress-alleviating rhizobacterium. The EH2-5 symbiosis significantly enhanced a key ABA biosynthesis enzyme-related gene NCED3, dehydration responsive transcription factors DREB2A and NAC29 salinity stresses (100 mM and 150 mM). Moreover, the reduced expression of peroxidase (POD), superoxide dismutase (SOD), and catalase (CAT) by 16%, 29%, and 24%, respectively, and decreased levels of malondialdehyde (MDA) and hydroxy peroxidase (H_2_O_2_) by 12% and 23% were observed under 100 mM NaCl compared to non-inoculated plants. This study demonstrated that *Bacillus aerius* EH2-5, a melatonin-producing strain, not only functions effectively as a biofertilizer but also alleviates plant stress in a manner comparable to the application of exogenous melatonin. These findings highlight the potential of utilizing melatonin-producing microbes as a viable alternative to chemical treatments. Therefore, further research should focus on enhancing the melatonin biosynthetic capacity of EH2-5, improving its colonization efficiency in plants, and developing synergistic microbial consortia (SynComs) with melatonin-producing capabilities. Such efforts will contribute to the development and field application of EH2-5 as a promising plant biostimulant for sustainable agriculture.

## 1. Introduction

In 2021, global soybean (*Glycine max* (L.) Merr.) production exceeded 371 million metric tons, contributing significantly to the global food supply by providing over 25% of plant-derived protein (ourworldindata.org). Despite its importance, soybean growth is highly susceptible to environmental challenges, particularly soil salinization, which adversely affects plant morphology and development, ultimately leading to yield losses and diminished seed quality [1]. Compared to other staple crops such as wheat and rice, soybean is considered salt-sensitive, with salt stress reported to reduce yield by approximately 40% or even result in complete crop failure under severe conditions [2]. Salinity-induced stress leads to osmotic imbalance, ion toxicity, disrupted mineral nutrition, and a disturbed Na^+^: K^+^ homeostasis [3]. Among these effects, osmotic stress plays a critical role by triggering stress-responsive transcription factors and enhancing proline accumulation, which aids in maintaining cellular osmotic balance and strengthening plant tolerance to abiotic stress [4].

Salt stress represents a significant environmental constraint on plant growth and agricultural productivity. Elevated salinity levels induce osmotic imbalance, ion toxicity, and oxidative stress, all of which collectively hinder plant development and reduce crop yields [5]. To cope with such stress, plants have developed diverse adaptive mechanisms, including the maintenance of ion homeostasis, the synthesis of osmoprotectants, and the induction of stress-responsive gene networks. Among these, the activation of abscisic acid (ABA) signaling is a key physiological response to salt stress [6]. ABA contributes to enhanced stress tolerance by modulating gene expression, ion transport processes, and the cellular redox environment [7].

Melatonin (MEL), initially discovered in bovine pineal glands [8], is a multifunctional molecule known for its role in circadian rhythm regulation and nocturnal signaling in animals [9]. In plants, MEL has been detected in various organs—roots, stems, leaves, flowers, fruits, and seeds—across both monocot and dicot species such as apple, rice, and tomato, highlighting its conserved role in plant growth and development [10,11]. Melatonin (MEL) plays a vital role in enhancing plant resilience to abiotic stresses by regulating seed germination, photosynthesis, biomass accumulation, fruit maturation, and stress-responsive gene expression, particularly those involved in antioxidant defense [12,13]. Through its ability to activate ROS-scavenging systems, MEL functions as a stress-protective molecule and promising biostimulant for improving crop performance under adverse environmental conditions [14]. Several studies have demonstrated that exogenous melatonin treatment to crops alleviates salt-induced damage by reducing reactive oxygen species (ROS) levels and enhancing K^+^ retention in plants such as cotton (*Gossypium hirsutum*), rice (*Oryza sativa*), wheat (*Triticum aestivum*), and cucumber (*Cucumis sativus*) through the regulation of stress-responsive gene expression [15,16,17,18].

Plant growth-promoting (PGP) microorganisms have attracted considerable interest in sustainable agriculture for their ability to enhance crop productivity, facilitate nutrient uptake, and improve plant tolerance to abiotic stresses such as salinity, drought, and extreme temperatures [19]. These beneficial microbes support plant health through various mechanisms, including nitrogen fixation, mineral solubilization, phytohormone production, and the induction of systemic resistance, positioning them as effective alternatives or supplements to conventional fertilizers and pesticides. Among them, *Bacillus* spp. stands out as particularly effective bio-inoculants due to their capacity to form resilient endospores, enabling survival under harsh environmental conditions [20]. In addition to their robustness, *Bacillus* strains contribute to plant growth by fixing nitrogen, solubilizing phosphate and zinc, producing growth-regulating hormones, and alleviating stress effects. Their strong root-colonizing ability facilitates the secretion of antimicrobial compounds and resistance inducers, thereby protecting host plants against a broad spectrum of pathogens, including Gram-negative bacteria [21]. Within this genus, *Bacillus aerius* demonstrated notable PGP traits, such as indole-3-acetic acid (IAA) production, ACC deaminase activity, and biocontrol capacity, along with significant tolerance to abiotic stresses like salinity and drought [22]. These characteristics make *B. aerius* a promising candidate for microbial bio-inoculant development in stress-affected agricultural environments. For instance, *B. aerius* strain BKOU-1, isolated from forest soil, was shown to produce hydrolytic enzymes (cellulase, lipase) and suppress phytopathogens such as *Fusarium oxysporum* and *Macrophomina phaseolina*, promoting plant growth and disease resistance [23]. Similarly, an ACC deaminase-producing *B. aerius* strain enhanced growth and stress tolerance in *Carthamus tinctorius* (safflower), likely through ethylene regulation under salinity and drought conditions [24].

Melatonin-producing plant growth-promoting microorganisms (PGPMs) have gained attention for their ability to enhance plant tolerance to abiotic stresses by modulating antioxidant systems, ion balance, and hormone signaling [25,26]. Among them, *Bacillus aerius* has not been previously reported as a melatonin producer. In this study, we identified the melatonin biosynthesis potential of *B. aerius* and investigated its role in improving soybean tolerance to combined heat and salinity stress. This finding highlights *B. aerius* as a novel candidate for sustainable stress management in crops. For this, we hypothesized that the application of melatonin-producing strain EH151 would mitigate the detrimental effects of salt stress, thereby enhancing stress tolerance in soybeans. This study was designed with three primary objectives: (1) to isolate and evaluate a novel melatonin-producing plant growth promoting bacterium—we use a combination of high throughput whole genome sequencing, phytohormone measurements, expression profiling, and biochemical analyses to identify melatonin production by a novel *Bacillus aerius*; (2) to investigate the physiological and transcriptomic responses in soybean, focusing on antioxidant enzyme activity under salinity stress conditions; and (3) to elucidate the detailed molecular mechanisms by which the PGPB promotes soybean growth and development and provide protection against salinity stress. This research presents a novel approach by identifying new melatonin-producing microbes with PGP potential and evaluating their application as eco-friendly solutions for managing combined abiotic stresses. Moreover, this work may contribute to the development of salt-tolerant soybean cultivars and offer sustainable strategies to address climate change-induced food insecurity.

## 2. Result

### 2.1. Identification of Chosen Bacterial Strain

**Figure 1:**
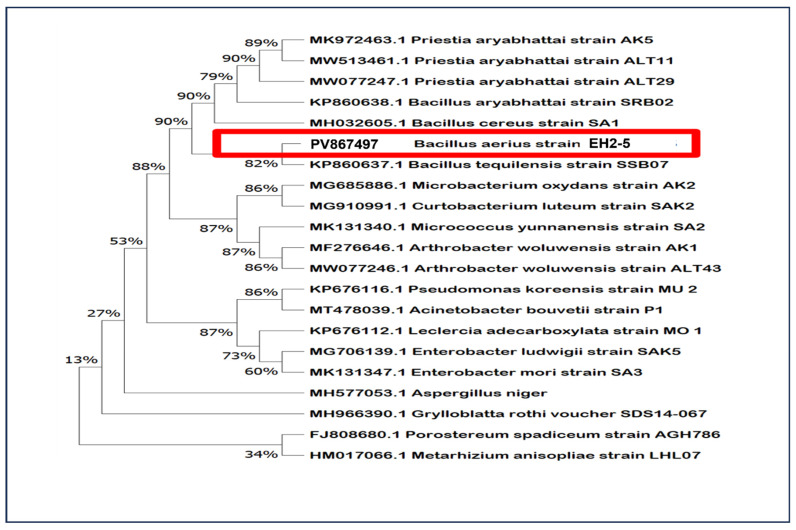
Phylogenetic tree based on 16S rRNA gene sequences showing the taxonomic position of *Bacillus aerius* strain EH2-5 (highlighted in red). The tree was constructed using the neighbor-joining method, and bootstrap values ≥ 50% from 1000 replicates are shown at the corresponding nodes. *Bacillus aerius* EH2-5 formed a clade with other well-characterized halotolerant or salinity-resistant bacterial strains, including members of the genera *Bacillus*, *Priestia*, *Microbacterium*, *Curtobacterium*, *Arthrobacter*, *Pseudomonas*, and *Enterobacter*. This phylogenetic proximity suggests a shared evolutionary adaptation to saline environments among the strains. *Aspergillus niger* and other distant taxa were included as outgroups.

A total of 751 rhizobacteria were isolated from the rhizosphere of tomato and pepper plants in the greenhouse. The chosen bacterial strain was isolated from the rhizosphere of tomato plants in the greenhouse. The 16S rDNA and gyrB gene sequencing and phylogenetic analysis disclosed that the selected PGPR strain (EH2-5) had a 100% similarity with the known sequences in GenBank and belonged to *Bacillus aerius*. In addition, the phylogenetic analysis of the EH2-5 strain using RAxML GUI v.1.3 software revealed its relevance to other strains of respective species, which have plant-growth-promoting (PGP) traits and salinity stress resistance (Figure 1). The 16S rRNA and gyrB gene sequences obtained in the present study have been submitted to the NCBI GenBank database and assigned the accession number PV867497.

### 2.2. Plant Growth-Promoting Traits of EH2-5

**Figure 2:**
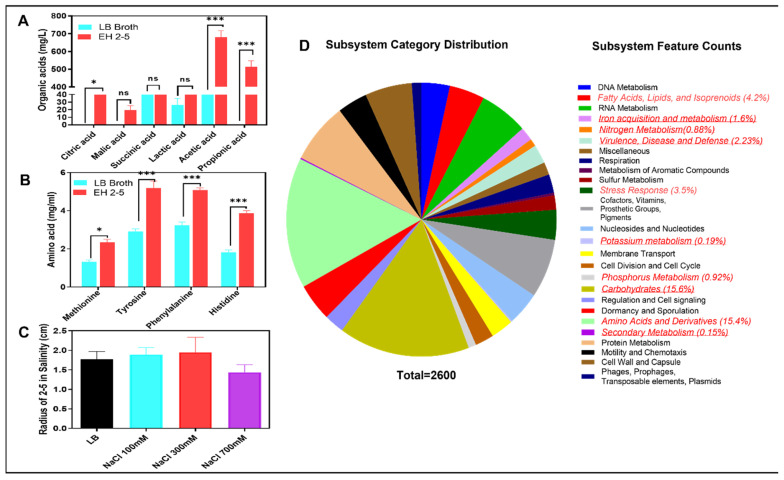
Metabolite production and genomic traits of *Bacillus aerius* strain EH2-5 under salinity conditions. (**A**,**B**) Quantification of beneficial metabolites produced by EH2-5: organic acids and amino acid derivatives involved in salinity tolerance and plant growth promotion. (**C**) Colony diameter of EH2-5 grown under salinity stress (150 mM NaCl), showing its halotolerant capacity. (**D**) Functional gene annotation of EH2-5 based on RAST analysis. The pie chart displays the relative abundance of annotated gene categories, with significant portions involved in carbohydrate metabolism (15.6%), amino acids and derivatives (15.4%), fatty acids and lipids (4.2%), stress response (3.5%), and virulence/disease defense (2.23%). Genes related to nitrogen (0.88%), phosphorus (0.92%), potassium (0.19%) metabolism, and iron acquisition (1.6%) highlight the strain’s genomic potential to confer abiotic stress tolerance and promote plant growth. Data represents the means of at least three independent biological replicates. Error bars represent standard deviations. Error bars represent standard deviations. Means were compared using Tukey’s post hoc test. * indicates *p* < 0.05, ** indicates *p* < 0.01, *** indicates *p* < 0.001, and ns: not significant.

EH2-5 exhibited an innate ability to tolerate high salt-induced stress. When cultured on standard LB medium supplemented with NaCl, the colony diameter of EH2-5 remained relatively unaffected even at elevated salt concentrations of 100 mM and 300 mM, indicating its halotolerant nature. When cultured on LB media supplemented with 100 mM and 300 mM NaCl, the colony diameter of EH2-5 increased by 6.3% and 9.8%, respectively, compared to that on normal LB medium (Figure 2C). Although the colony diameter decreased under 700 mM NaCl compared to the control, EH2-5 was still able to grow, indicating its strong salt tolerance even under extremely high salinity conditions. In addition, the plant-growth-promoting (PGP) traits of EH2-5 were confirmed through its ability to produce various amino acids and organic acids. Although standard LB medium inherently contains several amino acids and organic acids, comparative analysis between non-inoculated LB medium and EH2-5-inoculated LB medium revealed a substantial increase in metabolite production following EH2-5 inoculation. Specifically, methionine, tyrosine, phenylalanine, and histidine levels were 77%, 79%, 57%, and 111% higher, respectively, in the EH2-5-inoculated medium, indicating that EH2-5 has the capacity to synthesize these amino acids (Figure 2A). Similarly, levels of organic acids also increased markedly. Compared to the non-inoculated control, the EH2-5-inoculated medium showed dramatic increases in citric acid, malic acid, and propionic acid, while succinic acid increased by 49%, lactic acid by 304%, and acetic acid by 126% (Figure 2B).

The salinity resistance of EH2-5, along with its ability to produce beneficial plant growth-promoting (PGP) compounds such as amino acids and organic acids, is further supported by whole-genome sequencing (WGS) analysis (Figure 2D). The functional annotation of the EH2-5 genome using the RAST server revealed the presence of several gene categories associated with PGP and stress tolerance. Specifically, genes related to stress response accounted for 3.5%, amino acids and derivatives for 15.4%, fatty acid metabolism for 4.2%, carbohydrates for 15.6%, iron acquisition for 1.6%, nitrogen metabolism for 0.88%, and genes related to virulence, and defense for 2.23%. These genomic features indicate that EH2-5 harbors a diverse set of genes that potentially contribute to enhanced plant growth and resistance to both abiotic and biotic stresses.

### 2.3. Melatonin Producing Ability of EH2-5

**Figure 3:**
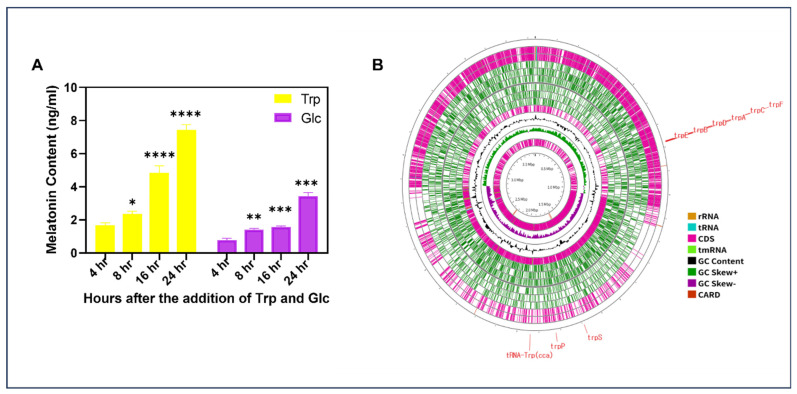
Tryptophan-derived melatonin biosynthetic pathway and genomic organization of *Bacillus aerius* EH2-5. (**A**) Melatonin levels were measured at 4, 8, 16, and 24 h in media supplemented with either tryptophan (yellow color)- or glucose (purple color)-spiked LB media to find the precursor compound of melatonin. (**B**) Circular genome map of *Bacillus aerius* EH2-5 showing the whole genome sequence and Proksee annotation of *Bacillus aerius* EH2-5. The identified tryptophan biosynthetic gene cluster (*trpE, trpD, trpC, trpB, trpA, trpF, trpS, trpP*) and tryptophan-specific tRNA genes (e.g., tRNA-Trp (cca)) are labeled in red, indicating a complete and active tryptophan biosynthesis module supporting *Bacillus aerius* EH2-5′s melatonin production potential. Data represents the means of at least three independent biological replicates. Error bars represent standard deviations. Means were compared using Tukey’s post hoc test. * indicates *p*  <  0.05, ** indicates *p*  <  0.01, *** indicates *p*  <  0.01, and **** indicates *p*  < 0 .001.

To determine whether glucose or tryptophan serves as the precursor for melatonin biosynthesis in strain EH2-5, LB medium was supplemented with either 1 mg/mL of tryptophan or glucose, inoculated with EH2-5, and sampled at 4, 8, 16, and 24 h post-inoculation for melatonin quantification. Comparisons between the tryptophan- and glucose-supplemented media revealed that melatonin production was significantly higher in the tryptophan-supplemented medium (Figure 3A). Specifically, in the presence of tryptophan, melatonin concentration reached 1.677 ng/mL at 4 h post-inoculation, whereas in the glucose-supplemented medium, melatonin levels reached only 1.58 ng/mL at 16 h. The highest melatonin concentration in the tryptophan-supplemented medium was 7.438 ng/mL at 24 h, while the glucose-supplemented medium reached a maximum of 3.434 ng/mL at the same time point. These findings indicate that EH2-5 utilizes tryptophan as a precursor for melatonin biosynthesis, likely following a tryptophan-dependent pathway similar to previously characterized melatonin synthesis routes. This conclusion is further supported by whole-genome sequencing (WGS) analysis, which revealed the presence of multiple tryptophan biosynthesis and metabolism-related genes in EH2-5, including trpP, trpS, trpE, trpB, trpD, trpA, and trpC (Figure 3B).

### 2.4. WGS Information of Strain EH2-5

Computational PROKSEE annotation revealed that EH2-5 has an innate ability to produce several secondary metabolites from different genomic regions. The list of genes in the EH2-5 genome that are important and might play a vital role in the synthesis of metabolites for the soybean plant–soil–salinity–EH2-5 interactions to regulate the metabolic pathways is listed in Table 1. The discussion section mainly focuses on a few probable genes of the EH2-5 genome that represent the important functions correlated with the microbe metabolite assay and plant physiology. The whole genome sequencing of EH2-5 revealed the presence of multiple genes associated with plant-growth-promotion (PGP) and salinity stress tolerance. Genes related to zinc uptake and regulation, such as znuA, znuB, znuC, and zur, as well as nitrogen metabolism genes, including ptsN, aspA, and nrgB, were identified. Several tryptophan biosynthesis and transport genes, such as trpA, trpB, trpP, and tspO, were also present. Phosphate metabolism genes, including phoH and pstS, were detected, suggesting potential involvement in phosphate solubilization or transport. In addition, siderophore-related genes (yusV, yfhA, yfiY), linked to iron acquisition, were found. A broad array of stress-related genes, such as dnaK, dps, treA, cspB, srkA, and copZ, was also present, indicating that EH2-5 possesses genomic features contributing to salinity and general abiotic stress resistance.

### 2.5. EH2-5 Improves Plant Growth and Development

**Figure 4:**
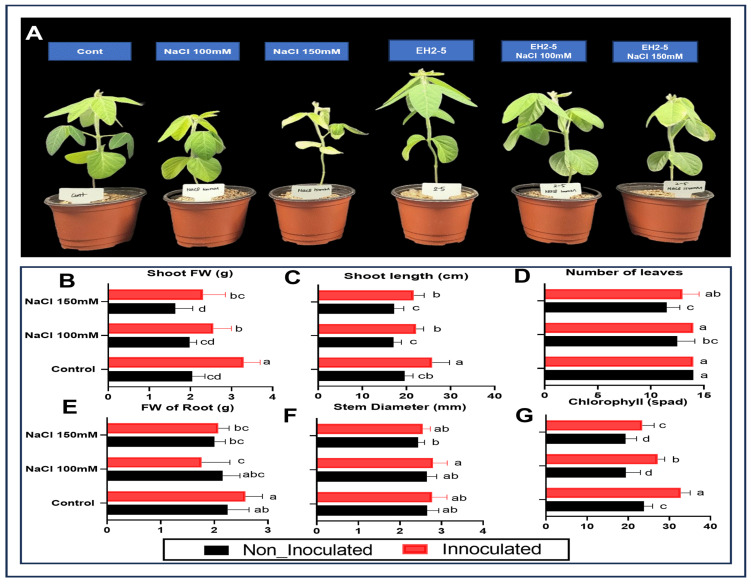
Phenotypic and physiological response of soybean seedlings to *Bacillus aerius* EH2-5 under salt stress conditions. (**A**) Representative images of soybean seedlings grown for 10 days under control (no salt), 100 mM, or 150 mM NaCl conditions, with or without EH2-5 inoculation. Plants treated with EH2-5 under 150 mM NaCl exhibited improved shoot growth, leaf development, and overall vigor compared to uninoculated controls under the same stress conditions. (**B**–**G**) Morphometric analysis of growth parameters, including shoot fresh weight, shoot length, number of leaves, root fresh weight, stem diameter and chlorophyll. Values are shown as the means ± SD (*n* = 12) and significant differences at *p* < 0.05 (Tukey test) are indicated by different lowercase letters above the columns.

This study investigated the growth-promoting and salinity stress-alleviating effects of strain EH2-5 in soybean seedlings. Two weeks after inoculating EH2-5 into the seedlings, salinity stress was applied at two concentrations (100 mM and 150 mM NaCl). As shown in Figure 4A, visual differences in growth were observed between non-inoculated control plants and those inoculated with EH2-5, under both non-stress and salt stress conditions (Figure 4A). In particular, EH2-5-inoculated plants showed reduced salt-induced damage compared to the control, with the protective effect being more pronounced under 150 mM NaCl, where stress severity was greater. Notably, shoot fresh weight in EH2-5-inoculated plants increased by 61% compared to non-inoculated controls. Under 100 mM and 150 mM NaCl stress conditions, inoculation with EH2-5 led to increases in shoot fresh weight by 29% and 41%, respectively, compared to the non-inoculated control (Figure 4B). Shoot length was also enhanced by 32% under non-stress conditions with EH2-5 and increased by 30% and 26% under 100 mM and 150 mM NaCl stress, respectively (Figure 4C). Similarly, shoot diameter and root fresh weight were consistently higher in EH2-5-inoculated plants regardless of salt treatment (Figure 4E,F). In terms of leaf number, EH2-5 inoculation resulted in 12% and 13% increases under 100 mM and 150 mM NaCl conditions, respectively, compared to the non-inoculated control (Figure 4D). Moreover, chlorophyll content, assessed by SPAD values, was 36% higher in EH2-5-inoculated plants under non-stress conditions, and showed increases of 40% and 21% under 100 mM and 150 mM NaCl treatments, respectively (Figure 4G). Taken together, these results demonstrate that EH2-5 significantly promotes plant growth and mitigates salt stress in soybeans.

### 2.6. EH2-5 Mitigates Salt Stress via Modulating of ROS with Antioxidants

**Figure 5:**
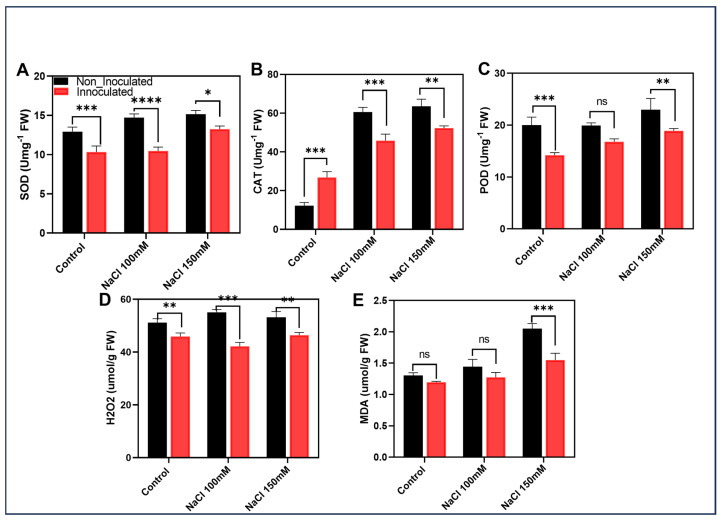
EH2-5 mitigates oxidative stress induced by salinity stress. (**A**–**C**) Antioxidant content: (**A**) SOD (superoxide dismutase), (**B**) CAT (catalase), and (**C**) POD (peroxidase) in leaves of soybean grown under normal and salinity stress conditions for 10 days after being inoculated with EH2-5. (**D**) Quantification of H_2_O_2_ in soybean leaves of plants subjected to NaCl-induced stress following EH2-5 inoculation. (**E**) Quantification of MDA. Data correspond to the means of three independent biological replicates. Error bars represent standard deviations. Means were compared using Tukey’s post hoc test. * indicates *p* < 0.05, ** indicates *p* < 0.01, *** indicates *p* < 0.01, **** indicates *p* < 0.001, and ns: not significant.

This study confirmed that the EH2-5 strain mitigates oxidative stress accumulation in plants under salinity stress, as evidenced by the quantification of intracellular hydrogen peroxide (H_2_O_2_) and malondialdehyde (MDA). Compared to the non-inoculated control, EH2-5-treated plants showed a 10% reduction in H_2_O_2_ under non-stress conditions. Under 100 mM and 150 mM NaCl stress, H_2_O_2_ levels in EH2-5-inoculated plants were reduced by 23% and 13%, respectively, relative to the non-inoculated treatments (Figure 5D,E). Similarly, MDA content decreased by 8% under control conditions with EH2-5 treatment. Under 100 mM and 150 mM NaCl stress, MDA levels in EH2-5-inoculated plants were 12% and 24% lower, respectively, than in non-inoculated plants. Furthermore, the activities of antioxidant enzymes under salt stress were less elevated in EH2-5-inoculated plants compared to the non-inoculated controls. Specifically, peroxidase (POD) activity was reduced by 16% and 18% under 100 mM and 150 mM NaCl, respectively. Catalase (CAT) activity was 24% and 18% lower, and superoxide dismutase (SOD) activity was reduced by 29% and 13%, respectively, in EH2-5-treated plants under the same stress conditions (Figure 5A–C). Collectively, these results indicate that EH2-5 alleviates salt-induced oxidative stress in plants by reducing the accumulation of oxidative stress markers and modulates the extent of antioxidant enzyme activation compared to non-inoculated controls.

### 2.7. Expression of Salinity-Responsive Genes During the Bacillus aerius EH2-5 Application

**Figure 6:**
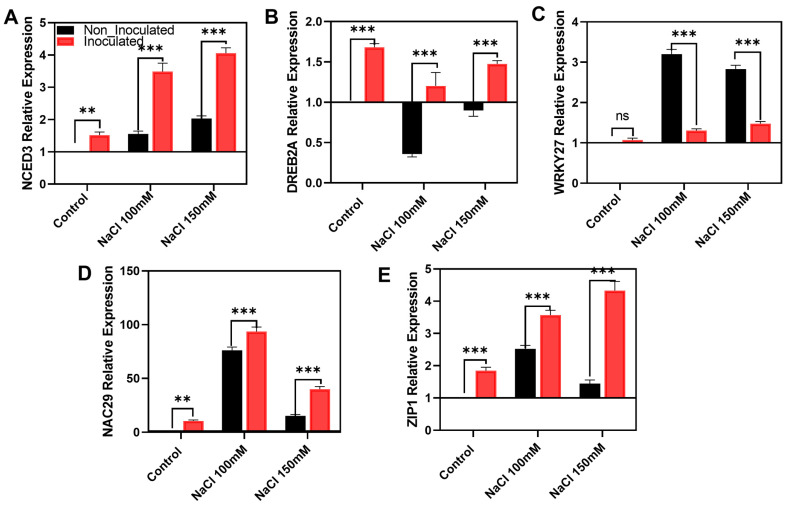
Relative expression of stress-responsive genes in soybean under salinity stress following inoculation with *Bacillus aerius* EH2-5. Quantitative real-time PCR analysis was performed to assess the transcript levels of key genes involved in (**A**) abscisic acid biosynthesis (*GmNCED3*), (**B**–**D**) dehydration and salinity response (*GmDREB2A*, *GmWRKY27*, *GmNAC29*), and (**E**) metal ion transport (*GmZIP1*). The upregulation of *GmZIP1* suggests a possible role for zinc homeostasis in stress adaptation facilitated by *Bacillus aerius* EH2-5. Data are presented as mean ± standard error (*n* = 3), normalized against internal control genes. Data corresponds to the means of at least three independent biological replicates. Error bars represent standard deviations. Means were compared using Tukey’s post hoc test. * indicates *p* < 0.05, ** indicates *p* < 0.01, *** indicates *p* < 0.001, and ns: not significant.

In addition, this study investigated the relative expression of various genes associated with salinity tolerance and dehydration stress. The expression of NCED3, a key gene in ABA biosynthesis and a well-known salinity-responsive marker, was significantly upregulated in EH2-5-inoculated plants. Specifically, under 100 mM and 150 mM NaCl stress, NCED3 transcript levels increased by 124% and 100%, respectively, compared to non-inoculated controls (Figure 6A). Also, the expressions of salinity-related transcription factors DREB2A, WRKY27, and NAC29 are examined (Figure 6B–D). The dehydration-responsive transcription factor DREB2A exhibited enhanced expression in EH2-5-treated plants, with increases of 236% and 64% under 100 mM and 150 mM NaCl stress, respectively, relative to non-inoculated plants. In contrast, the expression of WRKY27, a known negative regulator of salt and drought tolerance, was reduced by 59% and 48% under 100 mM and 150 mM NaCl, respectively, in EH2-5-inoculated plants compared to non-inoculated. Moreover, the expression of NAC29, a positive regulator of salinity tolerance, increased by 23% and 163% under 100 mM and 150 mM NaCl, respectively, in EH2-5-inoculated plants compared to non-inoculated. Finally, the expression of ZIP1, which facilitates zinc transport from plant roots to symbiotic bacteria in nodules, was upregulated by 42% and 200% under 100 mM and 150 mM NaCl, respectively, in response to EH2-5 inoculation (Figure 6E). Also, the upregulation of GmZIP1 suggests a possible role for zinc homeostasis in stress adaptation facilitated by *Bacillus aerius* EH2-5. These results suggest that EH2-5 not only enhances the expression of transcription factors and ABA biosynthetic genes involved in salinity tolerance but also promotes the expression of nutrient transport genes that may improve the symbiotic efficiency between soybean roots and nodule-associated microbes under saline conditions.

## 3. Discussion

The deposition of salinity stress in soybeans might induce a food toxicity disaster in the future [27].Therefore, combating the phytotoxicity induced by salinity is an important aspect of sustainable agriculture. The microbes, with their interaction with nutrient elements in soybean, could play a key role in achieving this goal [28]. In the current study, we applied the plant growth-promoting *Bacillus aerius* EH2-5 to detoxify the salinity toxicity in soybean plants. In particular, this study aims to investigate the effect of *Bacillus aerius* EH2-5 on alleviating salinity stress in soybeans, based on the novel finding that this strain produces melatonin. While the plant growth-promoting (PGP) abilities of *Bacillus aerius* have already been demonstrated, this study is the first to report its ability to synthesize melatonin. This objective is further supported by previous studies showing that exogenous application of chemically synthesized melatonin can enhance plant growth and mitigate various biotic and abiotic stresses [29,30]. By combining these insights, the current study explores the potential of a melatonin-producing *Bacillus aerius* strain as a biological agent to improve plant tolerance under saline conditions.

### 3.1. WGS of EH2-5

Salinity stress-resistant microorganisms and the synthesis of their metabolites play a key role in plant-microbe interactions to assist cross-signaling and augment stress tolerance in crops. In this study, bioassay analysis showed that EH2-5 could produce free amino acids, organic acids, and melatonin and significantly resisted more than 700 mM NaCl. Based on WGS of *Bacillus aerius* EH2-5, its key genes such as dnaK, dnaJ, and csaA encode molecular chaperones that assist in proper protein folding and protection against denaturation under high-salt and heat stress conditions [31,32]. The presence of cold shock protein cspD and general stress proteins (dps, yceD, yocK) suggests a broad adaptive capacity for environmental fluctuations [33]. HemW, a heme chaperone, and fra, an iron chaperone (frataxin), are implicated in maintaining redox balance and metal homeostasis, which are often disturbed under saline conditions [34]. Additionally, ykoL encodes a putative stress response protein potentially involved in osmotolerance [33]. This stress resilience enables it to persist in the rhizosphere under challenging conditions, where it can continue to exert plant growth-promoting effects such as nutrient solubilization, hormone production, and rhizosphere colonization. In soybean cultivation, the application of such stress-adapted beneficial microbes may improve plant tolerance to salinity and help maintain productivity in suboptimal soils [35,36]. This is further supported by the observation that soybean seedlings treated with EH2-5 under salinity stress exhibited improved growth parameters, including higher fresh weight and chlorophyll content, compared to untreated seedlings (Figure 4).

### 3.2. EH2-5′S Melatonin Producing Ability and Its Potential Ability as Biofertilizer

Melatonin biosynthesis has recently emerged as a notable plant growth-promoting (PGP) trait among microorganisms, due to its pivotal role in regulating plant metabolism and enhancing tolerance to environmental stress [25]. Although melatonin was historically regarded as a compound produced primarily by animals and plants, growing evidence indicates that diverse microbial taxa, including bacteria and yeast, are also capable of synthesizing melatonin [37,38]. Confirmed melatonin producers include *Pichia kluyveri*, *Bacillus*, *Variovorax*, *Oenococcus*, and photosynthetic bacteria such as *Rhodospirillum rubrum* [39]. In addition, melatonin production has been reported in several lactic acid bacteria, such as *Lactobacillus* spp., *Bifidobacterium* spp., and *Streptococcus thermophilus*, further expanding the diversity of melatonin-producing microbes [40].

Beyond its physiological role in microbial stress adaptation and redox balance, microbial melatonin significantly influences plant–microbe interactions. Several melatonin-producing PGP bacteria have demonstrated the ability to improve plant performance under abiotic stress. For example, *Pseudomonas* 42P4 and *Enterobacter* 64S1 produced 402.9 ng/mL and 99.0 ng/mL of melatonin, respectively, in tryptophan-supplemented cultures, and enhanced drought tolerance in Arabidopsis thaliana by increasing endogenous melatonin levels and preserving photosynthetic pigments [41]. Likewise, *Bacillus safensis* EH143, which synthesized 35 ng/mL of melatonin, was shown to reduce salt and cadmium stress in soybean through antioxidant regulation and the activation of stress-responsive genes [26].

These findings underscore the agricultural potential of melatonin-producing microbes as natural biostimulants. By boosting plant stress resilience, modulating hormonal responses, and enhancing nutrient assimilation, these microbes provide an eco-friendly alternative to chemical melatonin applications. In line with these observations, the present study highlights *Bacillus aerius* EH2-5 as a melatonin-producing strain with potential for promoting soybean growth and salinity tolerance, offering a promising tool for sustainable crop management under abiotic stress conditions.

### 3.3. Effects of Melatonin-Producing Microbes on Plants: Antioxidants and Salinity-Related Gene Expression

The beneficial effects of exogenous melatonin in alleviating oxidative stress—primarily through reactive oxygen species (ROS) scavenging and by preventing excessive melatonin accumulation—have been well established [42,43]. In this study, the endogenous melatonin-producing capability of strain EH2-5 may have similarly contributed to lowering ROS levels, including malondialdehyde (MDA) and hydrogen peroxide (H_2_O_2_), in soybean seedlings by reducing the plant’s need to synthesize melatonin. Inoculation with EH2-5 also resulted in reduced expression of antioxidant enzymes such as catalase (CAT), superoxide dismutase (SOD), and peroxidase (POD), indicating that the generation of ROS induced by salinity and cadmium stress was mitigated, thereby lessening the requirement for an intensified antioxidant defense response. These findings are consistent with previous studies, showing that beneficial microorganisms help regulate antioxidant enzyme activity to maintain redox homeostasis under abiotic stress.

Additional evidence of EH2-5′s protective role is reflected in the transcriptional regulation of key stress-responsive genes, including GmNCED3, GmDREB2A, and GmNAC29 (Figure 6). The upregulation of GmNCED3 supports abscisic acid (ABA) biosynthesis under osmotic stress caused by salt and drought [44]. GmDREB2A and GmNAC29, which encode transcription factors involved in dehydration and salinity tolerance, also showed increased expression following EH2-5 inoculation, suggesting enhanced abiotic stress resistance in treated plants compared to non-inoculated controls [45,46]. Conversely, the expression of GmWRKY27, a known negative regulator of salinity and drought tolerance, was lower in EH2-5-treated plants under saline conditions [47]. Since the overexpression of GmWRKY27 has been associated with reduced stress tolerance, whereas its suppression improves resistance, the downregulation observed here further supports EH2-5′s role in enhancing stress resilience. Taken together, these results indicate that *Bacillus aerius* EH2-5 promotes ion balance and alleviates oxidative stress in soybean, thereby enhancing the plant’s tolerance to salinity and associated dehydration stress.

Although this study demonstrated the beneficial effects of melatonin-producing strain EH2-5 on plant growth and salinity stress mitigation, it remains unclear whether the bacterium successfully colonizes the rhizosphere and actively produces melatonin in situ, and if so, to what extent. Furthermore, the mechanisms underlying the transport of microbially produced melatonin into plant roots—such as potential transporters or uptake pathways—have not yet been elucidated. These gaps limit our ability to fully confirm the direct absorption of microbial melatonin by the plant. Nevertheless, it is worth noting that many studies evaluating plant growth-promoting rhizobacteria (PGPR) under stress conditions do not provide such mechanistic details [48,49,50]. Importantly, our results showed significant physiological and biochemical differences between the EH2-5-inoculated plants and both uninoculated and stress-only controls, indirectly supporting the functional role of EH2-5-derived melatonin in promoting stress resilience.

### 3.4. Prospects and Challenges of Using Engineered Melatonin-Producing Microbiomes in Agriculture

Engineered melatonin-producing microbiomes, developed through synthetic community assembly or genetic engineering, offer a promising strategy to counteract plant dysbiosis triggered by biotic factors (e.g., pathogens, herbivores, parasitic weeds) and abiotic stresses (e.g., salinity, drought, heavy metals), ultimately supporting crop resilience and productivity [51]. These genetically modified microbes can be designed to produce melatonin in response to environmental signals, providing targeted stress mitigation—similar to effects observed in other PGP-engineered strains. Although exogenous melatonin has demonstrated agricultural benefits, its high cost (about USD 101 per gram for ≥98% purity from Sigma-Aldrich, USA) makes large-scale application impractical, and low-cost alternatives often lack quality assurance. In contrast, melatonin-producing microbes enable a more sustainable and economical approach by generating melatonin in situ, in synchrony with plant stress responses. When integrated into synthetic microbial consortia, they can also enhance other beneficial traits such as nutrient mobilization and disease suppression, functioning as multifunctional bioinoculants. Nonetheless, important challenges remain, including understanding the survival, colonization dynamics, and ecological interactions of these engineered strains in the rhizosphere. Risks such as unintended gene transfer and the disruption of native microbial communities must be carefully evaluated. Regulatory hurdles and public concerns about genetically modified organisms further complicate field deployment. Therefore, advancing this technology will require the development of precise gene regulation systems, long-term field validation across different cropping systems, and robust biosafety assessment frameworks to ensure safe and effective implementation in sustainable agriculture.

## 4. Methods

### 4.1. Isolation of Plant Growth-Promoting Bacteria

In this study, a total of 751 bacterial strains were isolated from the rhizosphere soils of strawberry, oriental melon, and tomato plants cultivated in high-salinity greenhouse environments located in Gunwi, South Korea (36°06′38.2″ N, 128°38′38.4″ E). To assess their plant growth-promoting (PGP) potential, the isolates were subjected to a series of functional assays. Siderophore production, phosphate solubilization, and extracellular polymeric substance (EPS) production were evaluated using CAS-Blue agar, phosphate solubilization media, and EPS-inducing media, respectively. Auxin production was quantified using the Salkowski reagent under dark conditions, followed by spectrophotometric measurement at 530 nm.

Based on their PGP performance, 100 promising bacterial strains were selected for the further screening of melatonin production. Each strain was cultured for two days, after which the culture supernatants were collected via centrifugation and extracted with ethyl acetate (1:1 ratio), followed by vortexing. The organic layer was then concentrated using a rotary evaporator under reduced pressure. Melatonin levels in the extracts were quantified using a commercial ELISA kit (Enzo Life Sciences, Farmingdale, NY, USA) and spectrophotometric analysis.

From this screening, the top 10 melatonin-producing strains were selected and evaluated for their tolerance to saline conditions. Among them, the strain exhibiting the highest salinity resistance was identified through 16S rRNA gene sequencing as *Bacillus aerius*. The sequence information was deposited in the NCBI GenBank under accession number PV867497.

### 4.2. Whole Genome Sequencing of EH2-5

Whole genome sequencing of the isolate was conducted at the Next Generation Sequencing (NGS) Core Facility of Kyungpook National University in Daegu, Republic of Korea. Genomic DNA was extracted using the Wizard^®®^ Genomic DNA Purification Kit (Promega, Madison, WI, USA) in accordance with the manufacturer’s protocol. DNA concentration and purity were assessed using a Qubit 2.0 fluorometer and a NanoDrop OneC Microvolume UV–Vis Spectrophotometer (Thermo Fisher Scientific, Waltham, MA, USA). For long-read sequencing, libraries were prepared with the Native Barcoding Kit 24 V14 and sequenced on the Oxford Nanopore MinION Mk1C platform using an R10.4.1 flow cell (Oxford Nanopore Technologies, Oxford, UK). In parallel, short-read sequencing was performed using the Illumina MiSeq platform (Illumina, San Diego, CA, USA), according to the standard workflow.

Base calling was performed using Guppy (v4.4.1) on an Ubuntu 18 GPU system (GeForce GTX 1660), and sequencing reads with Phred scores below 7 were filtered out. De novo genome assembly was carried out using Flye (v2.9) with default parameters, specifying a genome size of 4.0 Mb (--nano-raw --genome-size 4.0). The resulting contigs were scaffolded using CSAR and polished with Polypolish. Genome annotation was completed with the NCBI Prokaryotic Genome Annotation Pipeline (PGAP, v6.5), confirming the genome as complete. Final annotation, feature identification, and genome visualization were performed using the PROKSEE web platform (https://proksee.ca/).

### 4.3. Quantification of Organic Acids, Free Amino Acids in EH2-5 Cultures

The *Bacillus aerius* EH2-5 was cultured in LB broth and incubated at 28 °C for 72 h. Following incubation, cultures were centrifuged at 1000× *g* for 10 min at 4 °C. The culture supernatants were obtained by centrifugation as previously described, followed by filtration through a 0.45 µm membrane filter (DISMIC-25CS, Advantec, Tokyo, Japan). Free sugars and organic acids in the filtrates were analyzed using high-performance liquid chromatography (HPLC, Waters Co., Milford, MA, USA), according to the method described by [52]. For free amino acid detection, the filtrates were mixed with 5% trichloroacetic acid and shaken for 2 h. The mixtures were then centrifuged, further filtered through 0.22 µm syringe filters (StarLab, Hamburg, Germany), and subsequently analyzed using an amino acid analyzer (L-8900; Hitachi, Tokyo, Japan) with an injection volume of 20 µL per sample.

### 4.4. Quantification of Melatonin in EH2-5 Cultures

Melatonin production by the selected microbial isolates was initially assessed using the Ultra-sensitive Melatonin ELISA Kit (Enzo Life Sciences, Farmingdale, NY, USA). For further validation, strain EH2-5 was cultured in LB broth supplemented with tryptophan and incubated at 28 °C for 4, 8, 16, and 24 h. Following incubation, cultures were centrifuged at 1000× *g* for 10 min at 4 °C. A 5 mL aliquot of each supernatant was extracted with an equal volume of ethyl acetate by vortexing. The organic phase was collected and evaporated to dryness using a SpeedVac concentrator (Thermo Fisher Scientific, Waltham, MA, USA). The resulting pellets were resuspended in 125 µL of 1× stabilizer solution and prepared in at least two dilutions.

The pretreated samples were then transferred to a 96-well Goat anti-Mouse IgG Microtiter Plate. Each well received 50 µL of 1× melatonin tracer followed by 50 µL of 1× melatonin antibody. The plate was sealed and incubated on a shaker at 200 rpm for 1 h at room temperature. After incubation, the wells were emptied and washed three times with 400 µL of wash buffer. Subsequently, 200 µL of melatonin conjugate solution was added to each well, and the plate was sealed and shaken at 300 rpm for 30 min. Following another washing step, 200 µL of TMB substrate solution was added to each well, and the plate was incubated for an additional 30 min at 300 rpm. Finally, 50 µL of stop solution was added to terminate the reaction, and absorbance was measured at 450 nm using a Multiskan™ GO UV/Vis microplate spectrophotometer (Thermo Fisher Scientific, Waltham, MA, USA).

### 4.5. Plant Materials and Experiments

#### 4.5.1. NaCl and EH2-5 Treatment to Soybean Plants

Soybean seeds (cv. Pungsan) were germinated, and uniform seedlings at the VC stage were transplanted into individual pots (outer diameter: 10.0 cm; an inner diameter: 9.2 cm; height: 8.8 cm; and bottom diameter: 6.7 cm.) filled with autoclaved sandy soil. The plants were maintained under greenhouse conditions with 70% relative humidity and a temperature of 25  ±  4 °C and irrigated with *Bacillus aerius* EH2-5 cultures according to the treatment schedule outlined in Table 2. Following the completion of EH2-5 treatments, the bacteria were allowed to acclimate and establish root associations over a 10-day period. Subsequently, the seedlings were subjected to salinity stress using 100 mM and 150 mM NaCl, applied at specified volumes and frequencies (Table 2). After the salt stress period, normal irrigation resumed for seven days, during which plant physiological data were recorded. Leaf and root tissues were harvested, flash-frozen in liquid nitrogen, and stored at −80 °C for downstream analyses (Table 2).

#### 4.5.2. Analysis of Antioxidant Enzymes

The concentration of hydrogen peroxide (H_2_O_2_) in the samples was quantified following the protocol by [53]. In brief, leaf tissues were homogenized in 5 mL of 0.1% trichloroacetic acid (TCA) and centrifuged at 12,000× *g* for 15 min. From the resulting supernatant, 1 mL was mixed with 1 mL of 1 M potassium iodide and 0.5 mL of 10 M phosphate buffer (pH 7.0). The absorbance was then measured at 390 nm, and H_2_O_2_ content was calculated using an extinction coefficient of 0.28 mM^−1^ cm^−1^ and expressed as mg^−1^ dry weight (DW). Malondialdehyde (MDA) and superoxide anion levels in the leaves were measured according to the procedure described by [54].

Catalase (CAT) activity was determined using the method of [55], which involves monitoring the decomposition of H_2_O_2_ by measuring the decline in absorbance at 240 nm. The reaction mixture contained 15 mM hydrogen peroxide and 50 mM potassium phosphate buffer (pH 7.0), to which 100 µL of enzyme extract was added. CAT activity was then calculated using an extinction coefficient of 40 mM^−1^ cm^−1^. Superoxide dismutase (SOD) activity was assessed based on its ability to inhibit the photochemical reduction in nitro blue tetrazolium (NBT) in the presence of a fluorescent dye, as described by [56]. One unit of SOD activity was defined as the amount of enzyme causing 50% inhibition of NBT reduction, measured at 560 nm [56]. Peroxidase (POD) activity was measured following the method of [57]. A 0.1 mL aliquot of the supernatant was added to a reaction mixture containing 1 mL of 2% H_2_O_2_, 50 mM phosphate buffer (pH 5.5), and 50 mM guaiacol. A blank containing only the phosphate buffer served as a control. POD activity was recorded as the change in absorbance at 470 nm per minute over a three-minute interval [57].

#### 4.5.3. Quantitative Real-Time PCR

Total RNA was extracted from the leaves of ten individual soybean plants using the MagMAX™ Plant RNA Isolation Kit (Thermo Fisher Scientific, Waltham, MA, USA), following the manufacturer’s instructions. The concentration and integrity of the isolated RNA were assessed using the Qubit 4.0 Fluorometer with the RNA IQ Assay and RNA HS Assay kits (Thermo Fisher Scientific, Waltham, MA, USA). Complementary DNA (cDNA) was synthesized from 10 µL of RNA (100 ng/µL) using the High Capacity cDNA Reverse Transcription Kit (Applied Biosystems, Foster City, CA, USA) under the following thermal conditions: 25 °C for 10 min, 37 °C for 2 h, and 85 °C for 5 min. The resulting cDNA was stored at −80 °C until further analysis and was subsequently normalized for quantitative PCR. Quantitative real-time PCR was performed using PowerUp™ SYBR Green Master Mix and the QuantStudio 7 Pro Flex Real-Time PCR System (Applied Biosystems, Foster City, CA, USA). Primers (forward and reverse) were synthesized by Azenta and used at a final concentration of 10 pM for each reaction (Table S1). The qPCR cycling conditions were as follows: initial denaturation at 94 °C for 10 min; 35 cycles of 94 °C for 45 s, 65 °C for 45 s, and 72 °C for 1 min; followed by a final extension at 72 °C. Gene expression was analyzed using the ΔCt method. The entire experiment was conducted in triplicate.

### 4.6. Statistical Analysis

All experiments were performed in triplicate, and results are presented as means ± standard deviation. Statistical significance among treatments was assessed by one-way analysis of variance (ANOVA), followed by Tukey’s multiple range test at significance levels of *p*  <  0.05, *p*  <  0.01, and *p*  <  0.001. All statistical analyses were carried out using GraphPad Prism software (version 8.01; GraphPad Software Inc., San Diego, CA, USA).

## 5. Conclusions

To the best of our knowledge, this study is the first to elucidate the melatonin biosynthetic pathway in EH2-5 and its potential function in alleviating salt stress in soybean. This was supported by the observed reduction in salinity-induced oxidative stress in EH2-5-inoculated plants, which was accompanied by a lower induction of antioxidant enzyme activities. Additional evidence came from transcriptomic analyses and the expression patterns of key genes related to abscisic acid (ABA) biosynthesis, as well as transcription factors involved in salinity and dehydration responses. Furthermore, whole-genome functional annotation of EH2-5 revealed multiple relevant metabolites and associated genes contributing to its plant growth-promoting traits. Taken together, these findings highlight EH2-5 as a promising candidate for the development of melatonin-based biofertilizers. Ongoing studies aim to further uncover the molecular and genomic mechanisms underlying microbial melatonin biosynthesis.

## Figures and Tables

**Table 1:** Functional annotation of the genes in the EH2-5 genome with their gene locations, gene names, gene products, and description.

PGP Trait-Related Genes		
PGP Iron Genes		
Locus tag	Gene	Product
AAHJMGKC_03269	yfhA_1	Putative siderophore transport system permease protein YfhA
AAHJMGKC_03698	yfiY_1	Putative siderophore-binding lipoprotein YfiY
AAHJMGKC_03792	yusV_1	Putative siderophore transport system ATP-binding protein YusV
AAHJMGKC_03835	yusV_2	Putative siderophore transport system ATP-binding protein YusV
AAHJMGKC_03868	yfiY_2	Putative siderophore-binding lipoprotein YfiY
AAHJMGKC_03869	yfiZ	Putative siderophore transport system permease protein YfiZ
AAHJMGKC_03870	yfhA_2	Putative siderophore transport system permease protein YfhA
PGP Zinc Genes		
Locus tag	Gene	Product
AAHJMGKC_00039	znuA	High-affinity zinc uptake system binding-protein ZnuA
AAHJMGKC_00646	znuC	High-affinity zinc uptake system ATP-binding protein ZnuC
AAHJMGKC_00647	znuB	High-affinity zinc uptake system membrane protein ZnuB
AAHJMGKC_00648	zur	Zinc-specific metallo-regulatory protein
AAHJMGKC_01314		putative zinc protease
AAHJMGKC_01620	zosA	Zinc-transporting ATPase
AAHJMGKC_02153		Zinc-type alcohol dehydrogenase-like protein
AAHJMGKC_02961	ftsH	ATP-dependent zinc metalloprotease FtsH
AAHJMGKC_03775	cadA	Cadmium, zinc and cobalt-transporting ATPase
AAHJMGKC_03856	sufU	Zinc-dependent sulfurtransferase SufU
PGP Nitrogen Genes		
Locus tag	Gene	Product
AAMGKC_02753	ptsN	Nitrogen regulatory protein
AAHJMGKC_03243	aspA_1	Aspartate ammonia-lyase
AAHJMGKC_03494	nrgB	Nitrogen regulatory PII-like protein
AAHJMGKC_037	aspA_2	Aspartate ammonia-lyase
**Tryptophan-related Genes**		
PGP Tryptophan Genes		
Locus tag	Gene	Product
AAHJMGKC_00025	tspO	Tryptophan-rich protein TspO
AAHJMGKC_00896	trpB	Tryptophan synthase beta chain
AAHJMGKC_00897	trpA	Tryptophan synthase alpha chain
AAHJMGKC_01846	trpS	Tryptophan--tRNA ligase
AAHJMGKC_01971	trpP	Putative tryptophan transport protein
**Salinity Stress-related Genes**		
Locus tag	Gene	Product
AAHJMGKC_00045	dps	General stress protein 20U
AAHJMGKC_00050	yceD_1	General stress protein 16U
AAHJMGKC_00606	hemW	Heme chaperone HemW
AAHJMGKC_00609	dnaK	Chaperone protein DnaK
AAHJMGKC_00610	dnaJ	Chaperone protein DnaJ
AAHJMGKC_00962	cspD	Cold shock protein CspD
AAHJMGKC_01038	yocK	General stress protein 16O
AAHJMGKC_01058	csaA	Putative chaperone CsaA
AAHJMGKC_01232	fra	Intracellular iron chaperone frataxin
AAHJMGKC_01663	ykoL	Stress response protein Yk
AAHJMGKC_01943	ydaD_1	General stress protein 39
AAHJMGKC_01990	yhaX	Stress response protein YhaX
AAHJMGKC_02003	nhaX	Stress response protein NhaX
AAHJMGKC_02053	cspB	Cold shock protein CspB
AAHJMGKC_02185	yvgO	Stress response protein YvgO
AAHJMGKC_02205	yfkM	General stress protein 18
AAHJMGKC_02209	treA	Trehalose-6-phosphate hydrolase
AAHJMGKC_02210	treP	PTS system trehalose-specific EIIBC component
AAHJMGKC_02241	yflT_1	General stress protein 17M
AAHJMGKC_02254	yocM	Salt stress-responsive protein YocM
AAHJMGKC_02294	srkA	Stress response kinase A
AAHJMGKC_02498	cspC	Cold shock protein CspC
AAHJMGKC_02569	ydaG	General stress protein 26
AAHJMGKC_02570	ydaD_2	General stress protein 39
AAHJMGKC_02706	yceD_2	General stress protein 16U
AAHJMGKC_02707	yceD_3	General stress protein 16U
AAHJMGKC_02708	yceC	Stress response protein SCP2
AAHJMGKC_02794	surA	Chaperone SurA
AAHJMGKC_02980	ctc	General stress protein CTC
AAHJMGKC_03344	yflT_2	General stress protein 17M
AAHJMGKC_03483	yciC	Putative metal chaperone YciC
AAHJMGKC_03610	fliS	Flagellar secretion chaperone FliS
AAHJMGKC_03763	copZ	Copper chaperone CopZ
AAHJMGKC_03970	yugI	General stress protein 13
**Unclassified_Genes**		
Locus tag	Gene	Product
AAHJMGKC_00171	tpx	Thiol peroxidase
AAHJMGKC_00523	dhA	Glutathione-independent formaldehyde dehydrogenase
AAHJMGKC_00657	sodA	
		Superoxide dismutase [Mn]
AAHJMGKC_00679	gloC	Hydroxyacylglutathione hydrolase GloC
AAHJMGKC_00782	gloB_1	Hydroxyacylglutathione hydrolase
AAHJMGKC_01332	proS_1	Proline--tRNA ligase
AAHJMGKC_01728	ggt	Glutathione hydrolase proenzyme
AAHJMGKC_01822	kefB	Glutathione-regulated potassium-efflux system protein KefB
AAHJMGKC_01849	gsiC	Glutathione transport system permease protein GsiC
AAHJMGKC_02115	gsiD_1	Glutathione transport system permease protein GsiD
AAHJMGKC_02292	opuE	Osmoregulated proline transporter OpuE
AAHJMGKC_02485	cpo	Non-heme chloroperoxidase
AAHJMGKC_02603	kefG	Glutathione-regulated potassium-efflux system ancillary protein KefG
AAHJMGKC_02660	putR_1	Proline-responsive transcriptional activator PutR
AAHJMGKC_02662	putB	Proline dehydrogenase 2
AAHJMGKC_02778	proS_2	Proline--tRNA ligase
AAHJMGKC_02782	gloB_2	Hydroxyacylglutathione hydrolase
AAHJMGKC_03122	gloB_3	Hydroxyacylglutathione hydrolase
AAHJMGKC_03301	gloA	Lactoylglutathione lyase
AAHJMGKC_03376		Putative heme-dependent peroxidase
AAHJMGKC_03717	gsiD_2	Glutathione transport system permease protein GsiD
AAHJMGKC_03915	putR_2	Proline-responsive transcriptional activator PutR

**Table 2:** Experimental design.

Treatment	Detail
Cont	Irrigated with sterile distilled water only
NaCl 100 mM	Irrigated with 100 mM NaCl solution
NaCl 150 mM	Irrigated with 150 mM NaCl solution
EH2-5	Irrigated with EH2-5 broth culture
EH2-5 + NaCl 100 mM	Irrigated with 100 mM NaCl and EH2-5 broth culture
EH2-5 + NaCl 150 mM	Irrigated with 150 mM NaCl and EH2-5 broth culture

Note: Control (50 mL. Frequency = Once every day until completion of the experiment). EH2-5 (5 mL broth culture + 45 mL sterile H_2_O. Frequency = 3 times [Once every third day]). NaCl (50 mL. Frequency = 3 times [Once every third day]).

## Data Availability

The data analyzed in this study are publicly available through the LOVD database.

## Supplementary Materials

The following supporting information can be downloaded at: https://www.mdpi.com/article/10.3390/ijms26167834/s1.
