# Supplementary material for: Melatonin-Producing Bacillus aerius EH2-5 Enhances Glycine max Plants Salinity Tolerance Through Physiological, Biochemical, and Molecular Modulation
Source: Int J Mol Sci. 2025 Aug 13;26(16):7834. doi: 10.3390/ijms26167834 (PMC12386687; doi:10.3390/ijms26167834)
Supplement: Supplementary file 1 [file ijms-26-07834-s001.zip › ijms-3766371-supplementary.pdf]

Supplementary Table S1.

Name	Forwad (5'→3')	Reverse (5'→3')
NCED3	5'-ATGGCGAGGAGTTTTCCGTTGAAGAAGA-3'	5'-ATGGCGAGGAGTTTTCCGTTGAAGAAGA-3'
DREB27	5'-GGAGAATCCTCGACTTGTCAATGTG-3'	5'-CATGTGATCCACAGCCTCATCCTTA-3'
WRKY27	5'-GTAACAACAGGTTCCAACCGTTCA-3'	5'-CTTCTGGTGATTCAGTTTTGGGATT-3'
NAC29	5'-ACGGATGAGGAACTGATTGTGT-3'	5'-TGGGTACTTCCTGTCTCTTGGA-3'
ZIP1	5'-ACCAACACCAACAACATCCA-3'	5'-TTGAATGCTCAGCAGCAACT-3'
ACT	5'-ATCTTGACTGAGCGTGGTTATTCC-3'	5'-GCTGGTCCTGGCTGTCTCC-3'
